# Instantaneous neural processing of communicative functions conveyed by speech prosody

**DOI:** 10.1093/cercor/bhab522

**Published:** 2022-02-08

**Authors:** Rosario Tomasello, Luigi Grisoni, Isabella Boux, Daniela Sammler, Friedemann Pulvermüller

**Affiliations:** Brain Language Laboratory, Department of Philosophy and Humanities, Freie Universität Berlin, 14195 Berlin, Germany; Cluster of Excellence ‘Matters of Activity. Image Space Material’, Humboldt Universität zu Berlin, 10099 Berlin, Germany; Brain Language Laboratory, Department of Philosophy and Humanities, Freie Universität Berlin, 14195 Berlin, Germany; Cluster of Excellence ‘Matters of Activity. Image Space Material’, Humboldt Universität zu Berlin, 10099 Berlin, Germany; Brain Language Laboratory, Department of Philosophy and Humanities, Freie Universität Berlin, 14195 Berlin, Germany; Berlin School of Mind and Brain, Humboldt Universität zu Berlin, 10117 Berlin, Germany; Einstein Center for Neurosciences, 10117 Berlin, Germany; Research Group ‘Neurocognition of Music and Language’, Max Planck Institute for Empirical Aesthetics, 60322 Frankfurt am Main, Germany; Department of Neuropsychology, Max Planck Institute for Human Cognitive and Brain Sciences, 04103 Leipzig, Germany; Brain Language Laboratory, Department of Philosophy and Humanities, Freie Universität Berlin, 14195 Berlin, Germany; Cluster of Excellence ‘Matters of Activity. Image Space Material’, Humboldt Universität zu Berlin, 10099 Berlin, Germany; Berlin School of Mind and Brain, Humboldt Universität zu Berlin, 10117 Berlin, Germany; Einstein Center for Neurosciences, 10117 Berlin, Germany

**Keywords:** communicative functions, electroencephalography (EEG), pragmatics, prosody, sensorimotor system

## Abstract

During conversations, speech prosody provides important clues about the speaker’s communicative intentions. In many languages, a rising vocal pitch at the end of a sentence typically expresses a question function, whereas a falling pitch suggests a statement. Here, the neurophysiological basis of intonation and speech act understanding were investigated with high-density electroencephalography (EEG) to determine whether prosodic features are reflected at the neurophysiological level. Already approximately 100 ms after the sentence-final word differing in prosody, questions, and statements expressed with the same sentences led to different neurophysiological activity recorded in the event-related potential. Interestingly, low-pass filtered sentences and acoustically matched nonvocal musical signals failed to show any neurophysiological dissociations, thus suggesting that the physical intonation alone cannot explain this modulation. Our results show rapid neurophysiological indexes of prosodic communicative information processing that emerge only when pragmatic and lexico-semantic information are fully expressed. The early enhancement of question-related activity compared with statements was due to sources in the articulatory-motor region, which may reflect the richer action knowledge immanent to questions, namely the expectation of the partner action of answering the question. The present findings demonstrate a neurophysiological correlate of prosodic communicative information processing, which enables humans to rapidly detect and understand speaker intentions in linguistic interactions.

## Introduction

In everyday social interactions, understanding the speaker’s intentions requires listeners to make assumptions about the communicative function beyond the literal meaning of utterances and words and signs they are composed of (Grice 1957; Wittgenstein 1965). A critical feature in spoken language is intonation or prosody that often serves as a medium to express the intended communicative purpose of the speaker (Bolinger 1986; Hellbernd and Sammler 2016). The audible melody—or intonation contour—of vocal pitch change or, in acoustic terms, the fundamental frequency (F0) variation is a main key to the interpretation of utterances (Bolinger 1986; Cruttenden 1997). Already at the earliest stages of language development, infants make use of vocal pitch to communicate their intentions, for example, in the one-word stage by marking single words as requests or complaints (Dore 1975; Prieto et al. 2012; Frota et al. 2014). Similarly, in adults’ conversation, the same linguistic utterance can convey different communicative intentions (e.g., criticisms, wishes, or suggestions) solely distinguishable by prosodic markers (Hellbernd and Sammler 2018, 2016).

It is widely accepted that specific prosodic cues correlate with distinct communicative functions. For instance, questions are characterized by a rising vocal pitch typically in the final part of the contour, in contrast to the falling pitch used to express statements (Lieberman 1967; Bolinger 1978; Srinivasan and Massaro 2003). Language development studies have reported that infants as young as 5–8 months are already able to distinguish between these two communicative functions based on prosodic cues (e.g., Frota et al. 2014). Further cross-linguistic work has shown that listeners can identify a question by the rising pitch level toward its end, even when perceiving languages not known to them (Chien et al. 2020; Gussenhoven and Chen 2000; see also Ohala 1994). Across languages, questions and statements are characterized by different syntactic (word order) and prosodic cues. However, the two features are not always independent; for example in German, a specific sentence form already comes with a strong bias toward one type of speech function and prosody (verb first for question and rising pitch, Ladd 2008). Such association of syntactic and prosodic cues is typically absent in Italian spoken language, where prosodic markers are often the sole feature determining the communicative functions of questions and statements (Agard and Di Pietro 1965; Fiorelli 1965; Chapallaz 1979; Maiden and Robustelli 2014). Merely by the terminal rising or falling pitch movement of a sentence like *stanno pulendo la casa*—“they are cleaning the house,” listeners are able to distinguish between questions and statements.

To date, a substantial number of neurophysiological research has focused on the role of voice pitch in sentence segmentation (prosodic boundary cues) during language processing (Pannekamp et al. 2005; Männel and Friederici 2009; Itzhak et al. 2010; Sammler et al. 2010; Bögels et al. 2011; Teoh et al. 2019), by primarily examining the closure positivity shift, an event-related potential (ERP) component characterized by a frontocentral distribution and indicating prosodic boundaries (Steinhauer et al. 1999). In contrast, other neurocognitive studies have focused on the role of prosody in expressing speaker’s emotional states (Ethofer et al. 2006; Schirmer and Kotz 2006; Alter 2011; Brück et al. 2011; Alba-Ferrara et al. 2012; Witteman et al. 2012; Grandjean 2021). Only a few functional magnetic resonance imaging (fMRI) studies (Sammler et al. 2015; Hellbernd and Sammler 2018) have investigated the effect of intonation on the listener in communicative function understanding. Sammler et al. (2015) reported the existence in the right hemisphere of a ventral auditory pathway along the superior temporal lobe and a dorsal auditory-motor one during the comprehension of single words that function as statement or question types. Functional interactions between auditory and “social” brain areas have also been documented in the processing of single words differing in prosody that express criticisms, wishes, and suggestions, whereby the latter areas are thought to signify the processing of information about theory of mind (ToM), including the attribution of mental states to oneself and others (Hellbernd and Sammler 2018). However, it is still unclear at which point in time the brain processes prosodic markers that convey different communicative functions and the underlying neural correlates. In the present study, we designed an electroencephalography (EEG) experiment to explore the effect of rising and falling intonation used to signal question and statement functions during the perception of well-matched Italian spoken sentences in the human brain.

In terms of linguistic pragmatic theories, prosodic markers are informative about the communicative function of sentences, that is, the illocutionary role or speech act type that motivates their use (Austin 1975; Searle 1979). The propositional semantic content of a linguistic utterance makes it clear what the communication is about, but the illocutionary role can be different even though the content is the same. For example, the sentence “You are looking nice” produced with a constant intonation, at the same pitch and loudness throughout, might be understood as a statement or assertion, whereas the same sentence produced with variable prosody, including strong amplitude modulation and F0 changes, seems best suitable for the communicative function of making a compliment. Likewise, prosodic cues may bias the listener toward a statement or question understanding.

A range of recent neurocognitive experimental studies have already reported brain correlates of specific speech acts and social–communicative interactions (Van Ackeren et al. 2016, 2012; Egorova et al. 2013, 2014, 2016; Bašnáková et al. 2014, 2015; Gisladottir et al. 2015, 2018; Tomasello, Kim, et al. 2019; Boux et al. 2021). In particular, several studies have focused on the fine-grained distinction between directive speech acts (e.g., requesting an object) and assertive speech acts (e.g., naming an object), in speech production and understanding, in spoken, written, and gestural modalities, and in increasingly lifelike natural social interactions (Egorova et al. 2013, 2014, 2016; Tomasello, Kim, et al. 2019; Boux et al. 2021). The results showed rapid emergence of different neurophysiological correlates for communicative function understanding of naming and request actions within 150 ms after the critical linguistic unit could first be perceived (Egorova et al. 2013, 2014; Tomasello, Kim, et al. 2019). Intriguingly, Tomasello and colleagues showed that the brain responds rapidly only when the speaker’s communicative intention and the propositional content are fully available, but not in communicative situations where, for instance, information about the communicative function of an action is available but without semantic content. The earliness of the neurophysiological differences between speech act types suggests that pragmatic information is processed very quickly and together with other linguistic information, including semantics and linguistic form (Tomasello, Kim, et al. 2019). This provides support for language models of parallel/simultaneous processing of different subtypes of psycholinguistic information (Marslen-Wilson and Tyler 1975; Marslen-Wilson 1987; Hagoort and van Berkum 2007; Pulvermüller et al. 2009; Shtyrov 2010; Strijkers et al. 2017).

The neurophysiological investigation of speech act processing led to an interesting side effect, the discovery of brain signatures that seem to be indicative of the processing of specific speech act types. One such example is the immediate (latency ca. 150 ms) activation of the hand-motor cortex in processing basic requests to hand over objects (Egorova et al. 2016; Tomasello, Kim, et al. 2019; see also Van Ackeren et al. 2016). A similar brain signature of requests has also been reported in speech act production (Boux et al. 2021). *Action prediction theory of communicative function*, as we prefer to term it, best explains the activation of the motor action system that has been reported for basic object-oriented verbal requests. Speech acts are typically embedded in trees of action sequences encompassing the actions that typically and regularly precede and follow it, and providing hints about the regular commitments and assumptions characterizing the speech act (Alston 1964; Hamblin 1970; Kasher 1987; Fritz and Hundsnurscher 1994; Fritz 2013). Requesting an object is typically linked with the partner’s action of grasping an object and handing it over to the speaker or with alternative actions of refusal or denial of the action. In contrast, the action sequence following an assertive speech act, such as naming, is typically not followed by an action, or denial or rejection of such a response. Hence, the relative enhancement of hand-motor region activation reflects the greater action affordance or the expectation of the partner’s actions in response to a request (Egorova et al. 2016; Van Ackeren et al. 2016; Tomasello, Kim, et al. 2019; Boux et al. 2021).

If we consider the activation of the motor cortex reported in previous studies on request understanding as a signature of the action sequence structure following a speech act, we may ask whether other speech acts of the same type would also show it (i.e., that a question calls for an answer or some other responses). Here we take questions as a test case, as according to standard speech act theories, questions are characterized by the intention to “request verbal information” from the listener, which makes them similar to other directive speech acts, such as requests or commands (Searle 1975; Searle and Vanderveken 1985). Because questions are intrinsically tied to the partner expectation of giving an answer, we predict early articulatory motor regions (e.g., areas controlling lip/tongue movements) to be more strongly activated for questions compared to statements (for which a similarly strong partner expectation is not present). This would mirror the typical action sequence of the partner, which is the preparation of a vocal response performed with the mouth to fulfill the speaker’s desire for information. This may differ from request understanding where the speaker expects an action performed with the hand, used for grasping and handing over the desired object to the speaker, which is reflected in the hand-motor region activation as reported previously (Egorova et al. 2016; Van Ackeren et al. 2016; Tomasello, Kim, et al. 2019; Boux et al. 2021). If so, such physiological similarities between request and question functions within the motor cortex would argue for similarities at the cognitive linguistic pragmatic level. In addition, we also expect ToM regions in temporoparietal cortex to be active for questions that have a richer commitment structure compared with statements, associated with the speaker’s desire to obtain the information, that the partner might know it and eventually be willing to follow the request. In contrast, assertives only commit the speaker to believe the stated proposition is true. This is also motivated by the fact that ToM regions have been shown to be active during request compared to naming understanding (Egorova et al. 2016) and also for other types of communicative functions (Ciaramidaro et al. 2007, 2014; Canessa et al. 2012; Spotorno et al. 2012; Bašnáková et al. 2014, 2015; Hellbernd and Sammler 2018).

In the present study, we asked the following questions: (i) At which point in time do brain signatures specific to speech act types emerge when communicative functions are solely conveyed by specific prosodic markers, that is, pitch contour in questions and statements? (ii) Would different spatial brain activation patterns be elicited by the speech acts of asking questions and making statements? And (iii) would the specific brain signatures of questions show similarities to those of object requests examined in previous studies, thereby yielding comparable results between different types of directive speech act types?

As our study had a main focus on the role of prosody in pragmatic understanding of social communicative actions, we also attempted to obtain clues about the role of prosodic features of speech specifically by separating those from the physical pitch contour changes that occur in nonvocal stimuli. Therefore, to isolate intonation patterns of language with content and syntactic structure from the effect of their physical acoustic features, subjects were also presented with low-pass filtered (LPF) speech signals and nonvocal musical sounds that mimicked the prosodic contours of the critical speech act sentences. Overall, while expecting distinct early brain activation between the different speech acts, where illocutionary force (or communicative function type) and speech content are clearly expressed, we predict the lack of similar differences for merely intonationally different stimuli in which such physical difference is divorced from semantic and propositional content—because the sound is either a nonvocal musical pattern or lacks speech content after LPF. This expectation was motivated by a previous study on speech act processing, where early speech act signatures could only be observed for stimuli that contained information about both illocutionary role and propositional content (Tomasello, Kim, et al. 2019).

## Material and Methods

### Participants

Twenty-six healthy right-handed volunteers (mean age 26 years; range 20–35; 13 females) took part in the study. All participants were monolingual native Italian speakers with normal or corrected to normal visual acuity and had no record of neurological or psychiatric disease. Participants were paid for taking part in the experiment, and their right-handedness was confirmed by the Edinburgh Handedness Inventory (Oldfield 1971) (mean laterality quotient ±79, 3.8 SE). The sample size was determined by a prior power analysis performed using G^*^power 3.1.9.7 (Faul et al. 2007). Based on Tomasello, Kim, et al. (2019), who investigated speech act processing by applying EEG, we assume an effect size of Cohen’s *d* = 0.29 with *α* = 0.05 and power = 0.8, resulting in a minimum sample size of 23 subjects. We recorded three more subjects in order to compensate for potential subject exclusion. Procedures were approved by the Ethics Committee of the Charité Universitätsmedizin, Campus Benjamin Franklin, Berlin, Germany. All participants signed an informed consent form prior to the start of the experiment.

### Stimuli and Procedure

Spoken sentences differing in intonation were used as experimental stimuli, along with control stimuli. Prosodic cues in the sentences indexed the communicative action of statements (e.g., describing an action) and questions (e.g., asking whether an action is being done). In the present study, we used standard Italian language, as it lacks any syntactic form (or rigid word order) to be used to mark yes/no questions. Thus, a sentence like *stanno pulendo la casa* “They are cleaning the house” can be understood as a statement or as a question depending solely on the sentence-final modulation of the F0 vocal pitch contour. We acknowledge that, in spoken Italian, there may be prosodic cues before the sentence-final word, but the modulation of the latter appears to be the most salient and clearest cue so that we focused on this feature in the present study.

**Figure 1:**
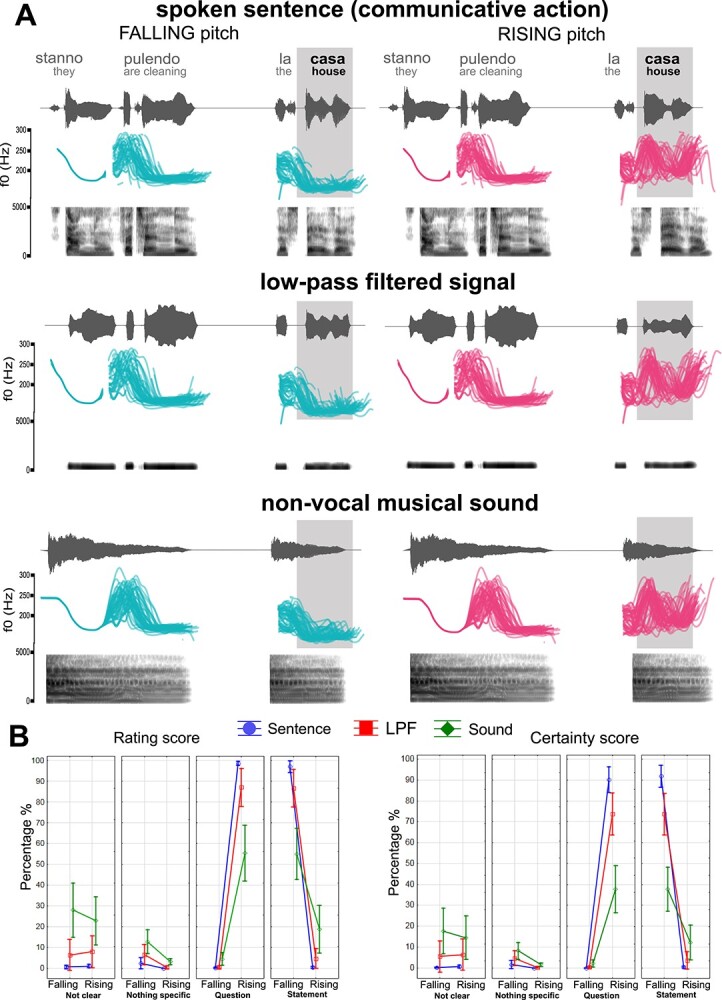
Example of the stimuli used and rating results. (*A*) Speech waveform and spectrogram plotted against time in gray for one stimulus example in each condition (spoken sentence, LPF signal, and nonvocal musical sound). Pitch contours in turquoise indicate falling pitch and in magenta rising pitch stimulus types presented to the subjects. Note the difference in pitch in the sentence-final word (shaded in gray). (*B*) Results of the stimulus ratings performed after the EEG recordings. The left and right panels show rating scores and certainty scores in percentages, respectively. Blue refers to spoken sentences, red to LPF signals and green to nonvocal musical sounds. Single diagrams illustrate the perceptual classification of rising and falling pitch stimuli as “question,” “statement,” “nothing specific,” or “not clear.”

A total of 110 sentences were recorded (44 100 Hz, 32 bit float, mono) from one female adult Italian native speaker who uttered third person sentences (e.g., *stanno mangiando la pasta*) as naturally as possible with either falling (statement) or rising (question) intonation on the sentence-final word (i.e., *pasta*) (see Lieberman 1967). To best match minor acoustic features before the sentence-final word, we cross-spliced the sentence material. The first two words (e.g., *stanno mangiando*) recorded with a neutral intonation were kept the same for both falling and rising pitch-ending sentences; 50% of the articles “*la”* preceding the final noun in a given sentence type were swapped and spliced into the respective other sentence type and vice versa. This way, it was made sure that the decisive difference in vocal pitch was confined to the sentence-final word, whereas the prosodic and other acoustic features of the rest of the sentence segment were controlled between the two stimulus types (see Fig. 1*A*). The length of each sentence was 3.5 s with a variable silent pause between 700 and 1000 ms prior to the article onset. The pause was necessary to separate the neurophysiological responses of the first sentence fragment from the critical final word and thereby allow for better signal to noise ratios and recordings uncontaminated by the preceding context (see e.g., Grisoni et al. 2021). It should be noted that hesitations and longer pauses typically occur during natural conversational interactions so that such speech sequences, including pauses might not have been perceived as unnatural. The sentence-final words included 44 disyllabic and 11 trisyllabic words for each rising and falling pitch condition, relating to everyday familiar objects. Finally, all the sentences were normalized to the same average sound energy and cleaned from background noise using Audacity 2.2.2 software (for the specific noise removal algorithm, see source code at https://sourceforge.net/projects/audacity/), which was also used for the splicing as described above. Ten sentences (five for each pitch stimulus type) were used as attention control items (or catch trials), in which the volume suddenly increased in the second word (i.e., the verb, *mangiando*). These stimuli were used to control if subjects were paying attention to the stimuli (additional information about the catch trials is given below).

To separate the contribution of syntactic and semantic information from that immanent to the F0 vocal pitch contour per se, a LPF was applied to every critical sentence with a 300 Hz cut-off and 30 Hz band smoothing. It was made sure that the content of the sentences was not understandable any longer after filtering, while the pitch contour of the sentence was still perceivable, sounding like “hummed speech.” Furthermore, we included a control condition in which the intonation patterns of the prosodic sentences were superimposed on a nonvocal musical sound (Piano Note A – 44 100 Hz, 32 bit float, mono, produced with the GarageBand synthesizer, a digital audio workstation). These manipulations were done using PRAAT 6.0.49 software (http://www.praat.org, Boersma 2001), resulting in a total of 330 stimuli, 110 in each condition (sentences, LPF signals, and nonvocal musical patterns). The mean fundamental frequency (Hz, pitch F0) and mean intensity (dB, root-mean-square [RMS] referred to the averaged intensity level of the sounds signals) of the sentence-final words in all sentences, and their corresponding control stimuli were extracted using customized scripts in PRAAT. Since the sentence-final word differed in duration, only the shortest common duration was analyzed, that is, 680 ms (see Table 1). Notice that we were interested in differences in early brain responses (within 200 ms upon the first detectable change) between falling and rising pitch conditions, that is, far earlier than the end of the shortest word. Similar results were obtained in an analogous acoustic analysis performed for the time window in which a significant EEG brain response difference was found, that is, between 68 and 118 ms (see Supplementary Table S1).

**Table 1:** Statistical comparison of the mean acoustic features of the sentence-final words

	Spoken sentence (communicative action)					
	Rising pitch		Falling pitch			
	Mean	SEM	Mean	SEM	*Z*-value	*P*
RMS (dB, intensity)	64.26	0.388	64.93	0.437	1.1529	0.248
Pitch F0 (Hz)	221.81	3.037	178.36	1.196	6.214	<0.001
	Low-pass filtered signal					
RMS (dB, intensity)	64.65	0.938	64.48	0.909	1.3685	0.171
Pitch F0 (Hz)	218.58	1.496	179.08	1.190	6.214	<0.001
	Nonvocal sound					
RMS (dB, intensity)	64.46	0.0157	64.47	0.0225	1.939	0.052
Pitch F0 (Hz)	218.41	1.5036	181.37	1.2304	6.205	<0.001

Note: The marginally significant difference in RMS (dB, intensity) in the nonvocal sound condition is due to the small variance within the stimuli (see the near-identical values of the mean and SEM).

The experiment consisted of three blocks, always starting with the nonvocal musical sound condition and followed, in counterbalanced order, by the LPF or speech act sentence conditions. The nonvocal condition was always presented as a first block to ensure that the understanding of rising and falling pitch sounds was not biased toward an interpretation as speech-like by the clear communicative functions of the speech conditions (sentence and LPF stimuli). Within each block, rising and falling pitch stimuli were presented in random order. Specifically, a list of rising and falling pitch stimuli was randomly generated for each condition (spoken sentence, LPF, and nonvocal tone), and then presented to each participant taking part in the experiment.

A fixation cross was always present on screen, to which the participants had to direct their eyes so as to minimize ocular movements that could alter the EEG signal. The presentation was controlled using E-prime 2.0 (Psychology Software Tools, Pittsburgh, PA). Each trial started with a stimulus presentation for 3500 ms and an interstimulus interval (ISI) that varied randomly between 1000 and 1500 ms. The experiment was conducted in the electrically and acoustically shielded chamber of the Brain Language Laboratory at the Freie Universität Berlin. Inside the chamber, a computer screen was used to present the fixation cross to the participants seated 80 cm away from the monitor. The acoustic stimuli were presented through high-quality headphones (AKG k271 Mkii) at a comfortable volume. The participant's task was to actively listen to all the stimuli (sentence, LPF, and nonvocal musical sound) and to try to understand what they might express. In addition, to check whether the participants were constantly attending to the stimuli, 10 stimuli (5 with falling and 5 with rising pitch) appeared in each of the blocks during “catch trials” pseudorandomly interspersed with the stimuli (i.e., between every 5 and 14 trials, a catch trial was presented). These catch trial stimuli contained a sudden change of the volume (+15 dB, 80 dB) in the second word. As a secondary task, subjects had to count how many of these “loudness deviants” they detected and reported the resulting number at the end of each experimental block. Brain responses of these catch trials were not analyzed. The entire EEG recording lasted approximately 30 min.

After the EEG recordings, participants were seated in front of a PC and were asked to rate the entire set of stimuli, which were presented in the same block order as in the EEG experiment. The procedure was as follows: subjects listened to each stimulus and had to respond to the question “What does this sound (sequence) communicate?” by choosing one of the four possible response options presented on screen: “probably nothing specific,” “wants to make a statement,” “wants to ask a question,” or “is not clear.” Upon responding, they had to rate on a continuous scale from 0 (unsure) to 100 (sure) how certain they were of their response. The ratings were controlled using psytoolkit (Stoet 2017). The recorded data were preprocessed before statistical analysis by first generating the frequency of categorization in the four options across items for each condition (sentence, LPF, and nonvocal musical sound) and then averaging for each subject. For better visualization, the resulting frequency scores were converted to percentages. For the certainty score, the values related to each response were first averaged across items, for each subject and then across all subjects, resulting in a certainty score in percent.

### Electrophysiological Recordings and Preprocessing

The EEG was recorded through 64 active electrodes embedded in a fabric cap arranged to the international 10-5 system (the green and yellow subsets of electrodes from the actiCAP 128Ch Standard-2; Brain Products GmbH, Munich, Germany) with the following modifications: the reference was moved from FCz position to the tip of the nose, the electrode occupying the PO10 position replaced the empty FCz position. The PO9 and FT9 electrode positions were reassigned as electrooculogram (EOG) channels placed below and above the left eye, respectively, and the FT10 electrode to the right outer canthus to measure the vertical and horizontal electrooculograms. All electrodes were referenced to the electrode placed on the tip of the nose. Data were amplified and recorded using the Brain Vision Recorder (version: 1.20.0003; Brain Products GmbH, RRID: SCR_009443), with a passband of 0.1–250 Hz, sampled at 500 Hz and stored on disk. Impedances of all active electrodes were kept below 10 KΩ.

The following preprocessing steps were carried out using EEGLAB 13.4.3b (Delorme and Makeig 2004). Data were bandpass filtered between 0.1 and 20 Hz by using the finite impulse response (FIR) filter. To obtain the vertical EOG, the difference between upper and lower left eye electrodes was calculated, and the horizontal EOG was computed from the average of the latter two minus the potential at the right outer canthus. Subsequently, the responses of individual EEG channels that contained no signal or significant artifacts after visual inspection were removed. Continuous EEG data were then epoched in large segments from sentence onset to 4 s post onset. Afterwards, independent component analysis (ICA) with the algorithm “runica” (Bell and Sejnowski 1995) was used to derive 41 components from the data. The derived ICA components that correlated with either vertical EOG or horizontal EOG with *r* > |0.3| were removed from the data, significantly reducing eye-related artifacts (Groppe et al. 2009; Hanna et al. 2014; Hanna and Pulvermüller 2014). On average, 2.6 out of 41 components were removed from each dataset. Subsequently, the noisy EEG electrode channels that had been removed before ICA were spherically interpolated back into the large segmented epoched data. The EEG data were then segmented into smaller epochs starting 320 ms before the noun onset (NO) and ending 700 ms after it. For baseline correction, the 200 ms prestimulus interval before the article onset was used, that is, from −320 to −120 ms. Epochs with signals exceeding −100 and 100 μV were discarded, and subjects with low signal-to-noise ratio (SNR < 2) were excluded in the final statistical analysis. Using this criterion, four data sets were rejected and therefore, data from 22 subjects entered the final EEG analysis. In this sample, the average trial rejection rate was 2.7%.

### Data Analysis

#### Stimulus Ratings

The data from the stimulus ratings performed by the subjects after the EEG experiment (for more details, see Methods section “*stimuli and procedure*”) were submitted to a 3 × 2 × 4 repeated-measures analysis of variance (ANOVA) with the factors condition (three levels: sentence, LPF, and nonvocal sound), pitch (two levels: falling and rising pitch), and response (four levels for the response alternatives “*probably nothing specific,” “wants to make a statement,” “wants to ask a question,”* and “*is not clear.’*” An analogous 3 × 2 × 4 repeated-measures ANOVA with the same factors was run on the certainty score data (0 unsure to 100 sure).

#### ERP Analysis

**Figure 2:**
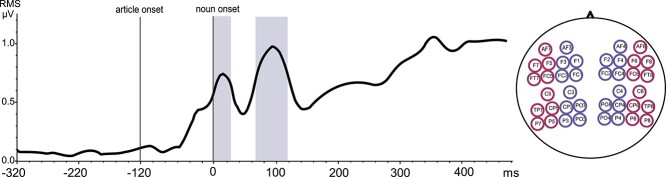
Selection of time windows for statistical analysis. RMS waveform computed across all EEG electrodes and averaged across all subjects and conditions. The zero point indicates onset of the critical noun, for example, “casa”; the preceding article (e.g., “la”) started at −120 ms. On the right-hand side, the electrode selection for the statistical analysis is shown.

To identify the peak latencies of the brain responses, the root mean square (RMS) waveform was calculated across all electrodes, subjects, and conditions (Fig. 2). The RMS allows us to determine the overall brain potential (μV) across the scalp as a function of time. Two time windows for analysis were defined using the full width at half maximum (FWHM) around the detected RMS peaks. To determine any differences in amplitude and peak latencies of the brain responses between falling and rising pitch sound sequences, we performed a series of repeated-measures ANOVAs. To this end, a large array of 40 fronto-central-parietal electrodes (AF7 AF3, AF4, AF8, F7, F5, F3, F1, F2, F4, F6, F8, FT7, FC5, FC3, FC1, FC2, FC4, FC6, FT8, C5, C1, C2, C6, TP7, CP5, CP3, CP1, CP2, CP4, CP6, TP8, P7, P5, P3, P1, P2, P4, P6, P8) were selected across the scalp. These were divided into anterior (frontal-central electrodes, AF, F, FC, FT labels) and posterior sites (central-parietal electrodes, C, CP, TP, P labels), and into left (odd electrode numbers) and right (even electrode numbers) hemispheres, and within each hemisphere, they were further divided into more peripheral (7, 5 6, 8) and central (3, 1, 2, 4) electrodes (see Fig. 2). Such electrode partitioning allows for a fine-grained investigation of topographical features of effects. A six-way ANOVA was performed with the factors time window (two levels: TW1 and TW2), condition (three levels: sentence, LPF, and nonvocal sound), pitch (two levels: falling and rising ending), and topographical factors anteriority (two levels: anterior and posterior), laterality (two levels: left and right hemisphere), and centrality (two levels: peripheral and central). Additional statistical analyses were performed on each time window and then separately for each condition (sentence, LPF, and nonvocal sound). Greenhouse–Geisser correction (Greenhouse and Geisser 1959) was applied when sphericity violations were found. Corrected *P*-values, along with epsilon (ε) values, are reported throughout. Partial eta-square (*η*_p_^2^) values are also stated, which is defined as an index of effect size (0.01–0.06 small, 0.06–0.14 medium, and >0.14 large; Cohen 1988).

### Source Estimation

We performed distributed source localization analyses to identify the cortical origin of the neurophysiological responses underlying the differences between falling and rising pitch stimuli. The method used for source estimation was the multiple sparse prior (MSP) technique, specifically the “greedy search” algorithm (Litvak et al. 2011), which had previously been used in our laboratory (e.g., Grisoni et al. 2017; Tomasello, Kim, et al. 2019). We used the structural MRI template included in SPM12 to create a cortical mesh of 8196 vertices, which was then coregistered with each subject’s electrode cap space using three electrodes as fiducials: FP1, TP9, and TP10. The volume conductors were constructed with an EEG (three-shell) boundary element model. Each response, within its respective time window, was then inverted for each subject, thereby constraining spatial source solutions uniformly across participants (Litvak and Friston 2008). Activation maps were then smoothed using a Gaussian kernel of FWHM 20 mm. Source averages and statistics were calculated at the group level and only in the time windows where significant effects between conditions were found in the statistical analysis described above.

To evaluate potential differences in source distribution between the conditions across the whole brain, we carried out voxel-wise paired *t*-tests. For the whole-brain source analysis, clusters that survived the threshold of *P* < 0.001 (uncorrected) were considered significant if they were larger than *k* > 10 voxels (i.e., cluster-extent based thresholding, Lieberman and Cunningham 2009; Woo et al. 2014). We further ran source analyses separately for the left and right hemisphere, to investigate the hemispheric involvement of linguistic prosody, which is a matter of debate among neuroscientists (e.g., Kreitewolf et al. 2014). In addition, we performed a third set of analyses in predefined regions of interests (ROIs) located in both hemispheres based on previous speech act, language processing, and intonation studies (Pulvermüller et al. 2006; Van Overwalle and Baetens 2009; Egorova et al. 2016; Tang et al. 2017). The ROIs included (i) left inferior frontal gyrus (IFG) found to be active during request speech act understanding (Egorova et al. 2014, 2016); (ii) left and right ventrolateral motor regions, where mouth movements are controlled (taken from a tongue movement localizer, and which have been shown to be active during silent articulation; Pulvermüller et al. 2006); (iii) left and right superior temporal gyrus (STG) shown to be active during intonational speech processing (Tang et al. 2017); and (iv) an area considered to be important for the processing of theory of mind (e.g., about the subject’s assumptions about views, intentions and feelings of others), that is, the right temporoparietal junction (rTPJ). The TPJ has been seen active in understanding other persons’ actions (Van Overwalle and Baetens 2009) and during recognition of several speech act types, including directives (Egorova et al. 2016; Van Ackeren et al. 2016; Hellbernd and Sammler 2018). ROIs were created with Marsbar 0.44 (MARSeille Boîte À Région d’Intérêt, SPM toolbox) as 20-mm-radius spheres (i.e., matching the FWHM of the smoothing parameter) centered on the coordinates documented by the previous studies listed above. These ROIs were then combined in a unique mask used as Explicit Mask in the voxel-wise paired *t*-test design. For the left and right hemispheres and ROI analysis, *P*-values were thresholded more conservatively (*P* < 0.05 after family-wise error [FWE] correction).

## Results

### Stimulus Ratings

The results of the repeated-measures ANOVA show a highly significant interaction between condition, pitch, and response (*F*(6, 78) = 23.31, ε = 0.22, *P* = 0.00004, *η*_p_^2^ = 0.64). This indicates a clear classification of both spoken sentences and LPF stimuli with falling and rising pitch endings as carrying statement or question functions, respectively. In contrast, the categorization of nonvocal sound was less clear and not significantly different in terms of their possible pitch-related functions (see Fig. 1*B*, left panel). A similar significant interaction between conditions, pitch, and responses was revealed by the ANOVA performed on the certainty scores (*F*(6, 72) = 17.66, ε = 0.56, *P* < 0.0001, *η*_p_^2^ = 0.59), showing high certainty in the classification of the critical spoken sentences and LPF stimuli as question or statement; in contrast, subjects were less certain in their categorization of the nonvocal sound stimuli (see Fig. 1*B*, right panel).

### Behavioral Results

Performance on catch trials, in which the participants had to detect a sudden volume change of the stimuli during the EEG experiment (for more details, see Methods section “*stimuli and procedures*”), was highly accurate (90%, SE = 0.6—27 out of 30 catch trials on average were correctly reported), demonstrating that the participants were paying attention to the stimuli presented via the headphones.

### ERP Results

**Figure 3:**
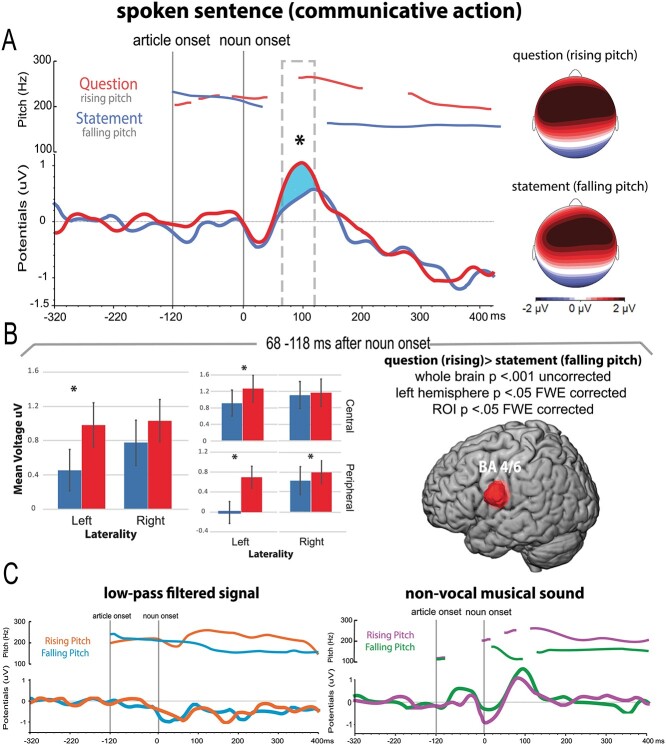
ERPs elicited by the different conditions along with the topographical and source analysis results. (*A*) Average pitch contours of sentence-final words in questions (red) and statements (blue) along with brain responses elicited by questions and statements in red and blue, respectively; the asterisk and highlighted blue area indicate the significant difference between conditions revealed by the statistical analysis between 68 and 118 ms after noun onset (NO). The topographical maps on the right show the scalp distribution of the ERPs evoked by rising and falling pitch. (B) The bar graphs depict the topographical comparison between question and statement ERPs in spoken sentences; error bars show standard error, asterisks indicate significant differences between conditions (Bonferroni planned comparison tests). The brain on the right illustrates the results of the source analysis, that is, brain regions showing stronger activity for questions than statements. (*C*) Average pitch contours and ERPs evoked by rising and falling pitch contours in LPF speech (orange and light blue) and nonvocal sounds (magenta and green).

The RMS waveform showing activation across all electrodes and conditions revealed peaks at 14 and 100 ms after after the critical noun onset (NO). Note that the first peak is likely due to the preceding determiner (article onset at −120 ms), whereas the second peak is (or includes) a P50/N100 to NO. Time windows around these peaks were defined by computing the FWHM, resulting in 0–48 ms and 68–118 ms after NO (see Fig. 2). Mean ERP amplitudes in these two data-driven time windows were fed into a six-way ANOVA (time window × condition × pitch × anteriority × laterality × centrality), which revealed a significant three-way interaction between time window, condition, and pitch (*F*(1, 21) = 3.4, *P* = 0.041, *η*_p_^2^ = 0.13). To further explore the differences between the conditions in each time window, we performed a five-way ANOVA for each time window separately, which revealed the following: the first time window (0–48 ms) did not show any significant interactions between the different factors (*F*(2, 42) = 1, *P* = 0.37), which is consistent with the assumption that this early peak probably reflects the brain's response to the preceding article, where no differences were expected. In contrast, the second time window (68–118 ms) revealed a significant interaction between condition, pitch, laterality, and centrality (*F*(2, 42) = 4.2, *P* = 0.020, *η*_p_^2^ = 0.16). To further disentangle the activation patterns within the different conditions in the second time window, we ran a four-way ANOVA (pitch × anteriority × laterality × centrality) for each condition separately. These statistical analyses showed no significant differences for the LPF signal (*F*(2, 21) = 0.1, *P* = 0.74) and the nonvocal musical condition (*F*(1, 21) = 1.9, *P* = 0.17). In contrast, the ANOVA for the sentence condition showed significant interactions between pitch and laterality (*F*(1, 21) = 4.7, *P* = 0.041, *η*_p_^2^ = 0.18), and pitch, laterality, and centrality (*F*(1, 21) = 6.3, *P* = 0.020, *η*_p_^2^ = 0.23) due to more positive ERP amplitudes for rising compared with falling pitch stimuli. Bonferroni-corrected planned comparisons (eight comparisons, corrected critical *P* < 0.00625) confirmed significant differences at left hemisphere electrodes (*P* < 0.001), specifically in the left peripheral (*P* < 0.0001) and central (*P* < 0.0001) electrodes and also in the right peripheral (*P* = 0.003) electrodes (see bar plots Fig. 3*B*). Additionally, to explore whether the different conditions (spoken sentence, LPF, and nonvocal sound) might differ in their neurophysiological responses at later time points, we performed the same ANOVA (condition × pitch × anteriority × laterality × centrality) described above in two later time windows (160–260 ms and 200–300 ms). The analysis showed no significant interactions between condition, pitch, and topography in either of these time windows.

### Source Estimation

To evaluate differences in source strength and spatial distribution of sources between falling and rising pitch sentence conditions, we performed voxel-wise paired *t*-tests (i) across the whole brain, (ii) separately for the left and right hemispheres, and (iii) in predefined ROIs, in the second time window (68–118 ms after noun onset) that showed significant differences in ERP amplitudes as described above. The early enhancement of question-related activity compared with statements (rising > falling pitch contrast) was associated with greater activity in motor regions in the left hemisphere (BA 4/6, *x* = −48, *y* = −8, *z* = 24, whole-brain contrast, *P* < 0.001 uncorrected; left hemisphere analysis: *P* < 0.05 FWE-corrected; ROI analysis—ventral motor (articulatory) cortex: *P* < 0.05 FWE-corrected, see Fig. 3*B* right panel and Table 2). The paired *t*-tests restricted to the right hemisphere and the other predefined ROIs did not show any significant differences at *P* < 0.05 FWE-corrected (right ventrolateral motor region *t*-value = 2.98, right temporoparietal junction *t* = 0.79, left inferior frontal gyrus *t* = 0.60, and superior temporal gyrus in the left hemisphere *t* = 1.72 and right hemisphere *t* = 1.46).

**Table 2:** Source analysis for the contrast question > statement in the time window 68–118 ms

Question > statement	*X*	*y*	*Z*	*t*-values	No. of voxels	*P*-value	Hemisphere	Brodmann areas	Regions
Whole-brain	−48	−8	24	4.23	876	0.0001 (uncorrected)	L	BA 4/6	Motor
Left hemisphere	−48	−8	24	4.23	95	0.043 (FWE corrected)	L	BA 4/6	Motor
ROIs	−48	−8	24	4.27	958	0.031 (FWE corrected)	L	BA 4/6	Motor

The table shows MNI coordinates, *t*-values, number of voxels for each cluster, *P*-values, hemisphere, and Brodmann and cortical region for each significant analysis.

## Discussion

The effect of prosody conveying different communicative functions of speech revealed early and distinct neurophysiological correlates in the listener’s brain. Perceiving the same spoken sentence ending with rising or falling pitch, and therefore signaling a question or statement function, elicited different ERP amplitudes and topographies at approximately 100 ms after the onset of the final critical word (see Fig. 3*A*). In contrast, rising and falling pitch contours in LPF speech signals and nonvocal musical conditions used to control for various acoustic and intonational features of the linguistic utterances did not show any neurophysiological differences (see Fig. 3*C*). Intriguingly, source localization revealed the greatest differences in cortical activation between questions and statements in left-hemispheric articulatory sensorimotor regions (Fig. 3*B*, right panel). These enhanced activations are best explained by the action-related nature of questions, in particular by the embedding of these communicative speech acts into other action schemas. A question is typically followed by the partner action of answering the question, implying the use of the speech production system. Speech production relates to activation of the articulatory sensorimotor system and corresponding brain regions. Therefore, the activation of the latter regions in question understanding is compatible with the view that understanding an utterance as a question is based on the anticipation of the typical partner reaction to a question, that is, answering. Below we will discuss the results in light of previous investigations on communicative actions, the role of prosody in language processing and linguistic pragmatic theories.

### Rapid Processing of Prosody Conveying Communicative Functions

The observed neurophysiological indices of statement and question functions at approximately 100 ms after the onset of the sentence-final word may reflect the neural processes that underlie the rapid discrimination of different communicative functions based on distinct prosodic cues. The current findings match well with previous investigations of brain correlates of linguistic pragmatic function processing in communication (Egorova et al. 2013, 2014; Tomasello, Kim, et al. 2019). In these previous studies, stronger brain activations were reported for directive as compared with assertive speech acts, already at approximately 150 ms after onset of single words appearing in communicative contexts where they functioned as tools for either naming or requesting; the context was provided either by previous verbal utterances (Egorova et al. 2013, 2014) or by gestures appearing simultaneously with words (Tomasello, Kim, et al. 2019). Notably, in our previous study, the fast dissociation between request and naming functions emerged only when the communicative functions were fully expressed through the combination of word and gesture, whereas gestures presented in isolation, that is, without referential information, showed a later neurophysiological dissociation (Tomasello, Kim, et al. 2019). Similarly, in the present work, the rapid brain response effects were only present when the communicative intention was conveyed through prosodic cues (expressing the speaker’s intention) and the semantic content of the utterance was available to the listener. In contrast, no brain response differences were found for the LPF signals, in which speech semantic content was lacking, but pitch intonation determining the communicative role (question vs. statement) was still perceived, as confirmed by the evaluation of the rating task. Likewise, no differences in brain responses were found in the nonvocal musical condition, where, in contrast to the LPF condition, the communicative role was entirely opaque, as revealed by the stimulus rating results (i.e., see Fig. 1*B* and the Results section). Hence, although listeners were able to recognize speech act types by means of prosodic modulation alone (see LPF rating results, and also Ohala 1994; Gussenhoven and Chen 2000), the present findings suggest that the brain discriminates the illocutionary role (speech act type) of communicative actions rapidly only if both pragmatic and lexical-semantic content (i.e., the requested information) is fully expressed by the speaker, which is in line with our previous EEG study on speech act processing (Tomasello, Kim, et al. 2019). This said we note that the brain indices of speech act function emerged 100 ms after the onset of the last word of the stimulus sentences, a point in time where these word final semantic units could perhaps not yet be recognized. However, our sentences ended in words that could be predicted to a degree from the context of their preceding sentence fragments so that it may well be that, together with the initial segment of the final word, sufficient evidence was available to at least make a good guess about the sentences’ meaning and function. For example, in the case of “they are cleaning the h…” (it: “stanno pulendo la c…”), the contextual evidence together with that for the word-initial phoneme may be sufficient to even safely predict the final lexical item (see e.g., Hagoort and Brown 2000). Assuming that a similar situation applies to many of our very common sentence stimuli, the speech act index in neurophysiology might correspond to the full processing of the perceived sentence meaning, which could be appropriately predicted at the onset of the final critical word. We note, however, that the issue of predictability of the critical word in prosodic–pragmatic information processing awaits more detailed future study, especially in the context of recent insights into the brain signatures of semantic prediction (Grisoni et al. 2021).

Notably, the fast and quick differentiation between the two communicative functions cannot be due to phonological, lexical, syntactic, or semantic aspects of the verbal material employed, as the same word sequence was used in the statement and question conditions, so that stimulus types only differed in their prosody. One may argue that systematic intonational differences between statements and questions may exist before the last word of Italian sentences. For example, D’Imperio (2002) argues that already at sentence onset, Neapolitan Italian statement and question intonations differ. However, looking at standard Italian, such early differences are less common (Agard and Di Pietro 1965; Fiorelli 1965; Chapallaz 1979; Maiden and Robustelli 2014) and in the output of our recordings from a native italian speaker, the clearest and most consistent intonational difference was in the sentences final noun phrase and in particular the terminal noun. Therefore, we matched exactly the sentence fragments up to the final noun, first by using the same sentence fragment (for both speech act functions) up to the final noun phrase and second by cross splicing half of the articles between the speech act conditions (see Methods). Thus, only the sentence final word differed in intonation. These methodological features enable us to draw firm conclusions on the early time course of prosody-based speech act comprehension. Within 100 ms of perceiving a prosodic difference between statement and question sentences, different brain indices of speech act functions were manifest. One may argue that our results may have been contaminated by the fact that the syntactic structure or sequence of lexical categories (verb-verb-article-noun) used may have biased listeners in favor of one of the speech acts. As pointed out in the introduction, many languages show habitual associations of statement and question functions with different word orders. However, we chose Italian spoken sentences precisely to avoid or minimize this problem, as Italian sentences used for statements and yes/no questions do not typically follow a rigid word order. Therefore, only acoustic prosodic cues determined the communicative role of question vs statement functions (Agard and Di Pietro 1965; Fiorelli 1965; Chapallaz 1979; Maiden and Robustelli 2014).

Altogether, the present results extend previous findings about the brain basis of pragmatic speech act processing by highlighting the critical role of prosody in conveying communicative functions in social interactions (Bolinger 1986; Hellbernd and Sammler 2016, 2018). In particular, it appears that linguistic–pragmatic information expressed through prosody along with lexico-semantic information is processed synergistically in the brain and very fast, that is, “within an instant” upon perceiving prosodic information along with the semantic information about the sentence content. Hence, these data make those language models somewhat problematic that still place pragmatic information access at the end of a language comprehension cascade, following phonetic, syntactic, and semantic processes in a cascading manner (Levelt 1993; Friederici 2002, 2011; Indefrey and Levelt 2004; Pickering and Garrod 2004, 2013). Instead, here we show an early time course of the neural signatures of the effect of prosodic markers conveying communicative pragmatic information together with semantic, syntactic, and phonological cues. This earliness is consistent with parallel models of language processing (Marslen-Wilson and Tyler 1975; Marslen-Wilson 1987; Pulvermüller et al. 2009; Shtyrov 2010; Strijkers et al. 2017).

### Brain Activation Patterns Indicating Communicative Action Understanding

Source reconstructions of the EEG responses revealed different cortical area activations for question compared with statement functions. The whole-brain contrast (question > statement) showed relatively stronger activation in the left articulatory-related motor cortex (tongue representation) (Pulvermüller et al. 2006; D’Ausilio et al. 2009; Strijkers et al. 2017), and this finding was further confirmed by the additional source analysis restricted to the left hemisphere and to predefined ROIs, where we applied strict statistical criteria (FWE correction, Fig. 3*B*). No differences were found in auditory cortices (left and right STG), left IFG, and right TPJ, underlining the spatial specificity of the effect. The specific motor region activation is best explained by what we would like to call the *action prediction theory of communicative function* processing, in which each specific speech act is defined as a set of possible partner actions typically following it (Alston 1964; Kasher 1987; Fritz 2013). In the case of question functions, the relative enhancement of the articulatory motor activation can be seen to underlie the partner’s expectation of uttering words in response to the speaker’s question. This evidence is consistent with previous neurocognitive studies documenting similarly enhanced sensorimotor activation in the hand motor region for requests to hand over objects compared to naming actions (Egorova et al. 2014, 2016; Tomasello, Kim, et al. 2019; Boux et al. 2021). Similarly, understanding indirect requests engaged regions implicated in action and motor control (Van Ackeren et al. 2012, 2016). Such specific hand motor area activation for request conditions may specifically reflect the processing of action knowledge about the partner response to a request, that is, the grasping of the requested object and handing it over to the speaker, which is performed by moving the hand. Here, we provide evidence that activation of sensorimotor regions is also present when processing questions, in which the predicted act of answering the speaker’s question by uttering words is reflected specifically in the articulatory motor region. Note that the fine-grained localization difference between requests and questions appears as particularly strong evidence for the idea that predicted partner actions that typically follow a speech act and differences between them are reflected in the mind and brain.

We would like to emphasize that the intrinsic link between speech acts and partner actions is, according to pragmatic–linguistic theory, not dependent on the immediately to be performed action. In our experiment and previous ones (Egorova et al. 2014, 2016; Tomasello, Kim, et al. 2019), only a few of the trials, and sometimes even no trials at all, had the relevant response action follow the critical speech act. The reason lies in the linguistic–pragmatic insight that there is a firm knowledge link between speech act function and partner response options (Alston 1964; Kasher 1987; Fritz 2013). It could be argued that such a link between speech act function and partner action, under special conditions, may be expected in response to LPF stimuli, for example the response, “Can you please repeat, I did not understand,” and thus similar articulatory motor activation as in the spoken sentence might be expected. However, an LPF sentence may result from various circumstances, including speech being used in an adjacent room or speech being processed by electronic devices, so that a partner response may be seen as the exception rather than the rule. Certainly, there is no similarly strong conventional link between LPF sentences and any specific type of verbal action as there is between questions and answers. Therefore, we do not believe that there is reason to predict any brain responses indexing partner action prediction in the case of LPF stimuli.

An alternative explanation for the stronger motor activation for questions (rising pitch) compared to statements (falling pitch) may be the possible involvement of the larynx area located next to the observed articulatory regions, which is involved in controlling vocal pitch (Brown et al. 2008; Dichter et al. 2018). However, the mentioned studies showed the larynx area’s involvement for prosodic processing in the production modality. Hence, a crucial question is whether the specific motor activation reflects (perceptual) tracking/mirroring of the pitch contour for phonological processing rather than question processing as described above or a mixture of both. We believe that, overall, the present set of results argues against a pure perceptual tracking origin because effects were found only in the spoken sentences, not in the LPF and not in the nonvocal sound conditions, although they both mimicked the prosodic contours of the sentences (i.e., rising and falling pitch). If our results reflected the involuntary laryngeal tracking of the rising pitch contours, similar effects should have been observed across all conditions, in particular in the LPF speech condition. Crucially, both LPF stimuli and spoken sentences were recognizable as speech stimuli, and their pitch contours were perceived similarly, as clearly revealed from the rating results (Fig. 1*B*). One may want to object against this point that the absence of a significant difference (here between LPF questions and statements) can never on its own provide a strong argument, as it represents a null result. However, our argument is not based on the absence per se but on the significant interaction (condition × pitch × topography), which shows, strictly speaking, that the question–statement difference is significantly more substantial in the speech than in the LPF conditions. This significant interaction provides relevant support for our claim. However, future studies with additional control conditions, additional recordings from the larynx, and spatially more precise neuroimaging methods (fMRI) are necessary to finally settle the issue of possible involvement of the laryngeal region and its interaction with the articulatory-motor regions in the processing of linguistic–pragmatic prosody during question and statement understanding.

The present brain data also contribute to the question of the hemispheric involvement into the processing of linguistic prosody during language understanding, which has been much debated among neuroscientists (Wildgruber et al. 2004; Witteman et al. 2011; Kreitewolf et al. 2014). In this respect, the data suggest that prosodic responses to spoken sentences containing linguistic–pragmatic information result in greater left hemispheric involvement, specifically, in the sensorimotor region (Broadman area 4/6) during perception of spoken sentences with a final rising pitch indicating question function. These findings are also consistent with a number of previous reports on the role of motor systems in signaling specific phonological, pragmatic, and semantic processes in speech production, comprehension, and prediction (Hauk et al. 2004; Pecher et al. 2004; Pulvermüller et al. 2005; Vukovic and Shtyrov 2014; Shtyrov et al. 2014; Dreyer et al. 2015, 2020; Tomasello et al. 2017, 2018; Tomasello, Wennekers, et al. 2019; Grisoni et al. 2021; Boux et al. 2021).

## Theoretical Implications and Future Directions

Finally, it is worth considering potential theoretical linguistic–pragmatic implications of our results and future directions. A theoretical linguistic debate addresses the core features of questions and their most appropriate classification into speech act groups. According to standard speech act taxonomy (Searle 1975; Searle and Vanderveken 1985), questions are characterized by the intention to request verbal information so that they can be classified, together with requests or commands, under the heading of directives (see also Sauerland and Yatsushiro 2017). However, other linguists argued that questions should be classified as distinct from directives, as an appropriate answer to a question is an assertion (Levinson 2012), which distinguishes questions from other requests. Others argue that a question is an incomplete statement (assertive) where the answer is the missing information (Goody 1978; for a review, see Wunderlich 1981), which in turn updates the speaker’s current state of information (i.e., common ground, Clark 1996; see also Sauerland and Yatsushiro 2017). These positions would move questions closer to the speech act category of assertives, whereas directives, including requests, would lack such incompleteness and information updating.

The current experiment may contribute to this theoretical debate of speech act classification by emphasizing the predominance of an action component in question processing. The most typical partner response to questions, the uttering of words for answering the questions and, thus, providing the requested information, seems to be manifest in terms of the locus of the articulatory motor activation. Given the physiological similarities between questions and other forms of directives (requests for objects, Egorova et al. 2016; Tomasello, Kim, et al. 2019; Van Ackeren et al. 2016), which all entail motor cortex activation, the current results argue for cognitive linguistic similarities at the neurobiological level between questions and other directives. Following the alternative perspective, where questions are seen as related to assertions, we would have expected a brain signature closer to that previously reported for statements or the frequently investigated speech act of naming, which have been shown to predominantly activate the left angular gyrus in the parietal lobe (Egorova et al. 2016); however, this was not the case in the present study. Therefore, the classification of questions as directives is consistent with the present neurophysiological data, favoring the Searlean perspective on a “bigger” category of directives. In future studies, it will be desirable to reinvestigate and possibly replicate the present results by exploring question and request functions within the same experiment and subjects to compare subtypes of directives and their brain correlates directly. In addition, it would be relevant to explore different types of questions, for instance, questions that are used to request information and rhetorical questions, which do not call for an answer response to the question but function in a similar way as an assertion (Koshik 2005).

In the present study, we found neurophysiological correlates of communicative function processing consistent with the action prediction theory, according to which the representations of predictable partner actions are part of the mental representation of speech acts. However, we want to emphasize that the sequence structure of question functions also relies upon other equally relevant pragmatic features that might be reflected at the cognitive and neural level. A feature that distinguishes questions from statements may also lie in the degree of complexity of social interactions where these speech act types are frequently used. One may argue that questions are typically used in more complex social situations as compared with statement situations (although counterexamples are, of course, easy to find). Furthermore, a complexity gradient seems to exist regarding the commitments in terms of the partner’s intentions and beliefs. Requests come with the commitment that the speaker intends to receive the requested information, that the partner can potentially follow the request, and that she or he is possibly willing to do so, whereas assertives only commit the speaker to believing the stated proposition. The difference in commitment structure also implies a difference in common ground, the shared knowledge intrinsically tied to communicative interaction (Clark 1996). Several neuroimaging studies have reported the involvement of an area in the TPJ that is frequently observed to be active in so-called ToM tasks or in social interaction processing generally (Saxe and Kanwisher 2003; Schurz et al. 2017). This area has also been reported active in several studies of social communicative understanding (Ciaramidaro et al. 2007, 2014; Canessa et al. 2012; Spotorno et al. 2012; Bašnáková et al. 2014, 2015; Egorova et al. 2014; Hellbernd and Sammler 2018). Specifically, the rTPJ, defined as the core area for ToM (Frith and Frith 2003; Gallagher and Frith 2003; Saxe and Kanwisher 2003; Amodio and Frith 2006), has been shown to be active during request compared to naming understanding (Egorova et al. 2016) and also during criticism, doubt, and suggestion understanding, whereby communicative functions were revealed by intonation (Hellbernd and Sammler 2018). A possible reason why the activation of the aforementioned areas was not detected in the present study might lie in the placement of the relevant cortical generators or in too low a spatial resolution of the EEG source analysis, to name only two possibilities. Therefore, a future experiment also using other localization tools (e.g., fMRI/ magnetoencephalography) should investigate a possible role of other areas, particularly the specific role of ToM areas and their interaction with the sensorimotor systems during speech act processing.

## Conclusion

The present neurophysiological (EEG) study examined the neural correlates of intonation features that discriminate between different communicative actions. The results showed that the difference between the communicative roles of questions and statements indicated by rising versus falling pitch contours is rapidly processed in the human brain. When listening to identical sentences differing only in intonation, brain responses differed clearly at 100 ms after the onset of the critical word whose intonation varied between the speech acts. This evidence highlights the crucial role of prosody in understanding the speaker intentions in language processing and, together with other recent evidence reviewed above, sits comfortably with the claim that pragmatic understanding processes are rapid and near-simultaneous with other facets of the language comprehension process. The control conditions, using LPF speech signals and nonvocal musical sound, failed to induce different brain responses between stimuli with rising and falling pitch contours, hence being consistent with the idea that the speech act differences cannot be explained by pure physical acoustic or pitch differences in isolation. The cortical origin of the larger activation of question compared with statement functions was located in sensorimotor regions that may carry information about the richer action knowledge immanent to questions, possibly related to the expectation of the partner answering the question, in turn. This interpretation is consistent with a directive component of question functions and a grouping of questions into the larger speech act category of directives. Altogether these findings highlight the ability of the human brain to instantaneously process different communicative functions conveyed by distinct prosodic markers along with the crucial importance of pragmatic processing in social communicative interactions.

## Supplementary Material

ProSA_SupplementaryMaterial_bhab522

LPF_pasta_Question_bhab522

LPF_pasta_Stastement_bhab522

Sound_pasta_Question_bhab522

Sound_pasta_Statement_bhab522

Speech_pasta_Question_bhab522

Speech_pasta_Statement_bhab522

## Notes

We would like to thank Manfred Krifka, Uli Sauerland, and Caterina Villani for comments and discussions and Verena Arndt, Verena Büscher, Giulio Perticari, and Milena Osterloh for their help with data collection and administration. *Conflict of Interest*: None declared.

## Data Accessibility

The datasets generated during the current study are available from the corresponding author upon reasonable request [RT].
