# Supplementary material for: Instantaneous neural processing of communicative functions conveyed by speech prosody
Source: Cereb Cortex. 2022 Feb 8;32(21):4885–901. doi: 10.1093/cercor/bhab522 (PMC9626830; doi:10.1093/cercor/bhab522)
Supplement: ProSA_SupplementaryMaterial_bhab522 [file prosa_supplementarymaterial_bhab522.pdf]

Supplementary material

Instantaneous neural processing of communicative functions conveyed by speech prosody

Rosario Tomasello[1,2]*, Luigi Grisoni[1,2], Isabella Boux[1,3,4] Daniela Sammler[5] and Friedemann Pulvermüller[1,2,3,4]

[1] Brain Language Laboratory, Department of Philosophy and Humanities, WE4
Freie Universität Berlin
14195 Berlin,
Germany

[2]Cluster of Excellence 'Matters of Activity. Image Space Material', Humboldt Universität zu Berlin
10099 Berlin,
Germany

[3] Berlin School of Mind and Brain, Humboldt Universität zu Berlin
10117 Berlin,
Germany

[4] Einstein Center for Neurosciences
10117 Berlin,
Germany

[5] Research Group 'Neurocognition of Music and Language', Max Planck Institute for Empirical Aesthetics,
60322 Frankfurt/M.,
Germany

*Corresponding author. Address for correspondence:

Brain Language Laboratory
Department of Philosophy and Humanities, WE4
Freie Universität Berlin
Habelschwerdter Allee 45
14195 Berlin, Germany
Tel.: +49 (0) 30 838 51984
Tomasello.R@fu-berlin.de
ORCID: 0000-0001-8414-2644

Supplementary material

	Spoken sentence (communicative action)					
	Rising Pitch		Falling Pitch			
	Mean	***SEM***	***Mean***	***SEM***	***Z-value***	***p***
RMS (dB, intensity)	69.43	0.72	69.30	0.62	0.2313	0.817
Pitch F0 (Hz)	250.07	5.84	178.10	3.40	5.118	<0.001
	**Low-pass Filtered signal**					
RMS (dB, intensity)	72.09	0.60	72.09	0.66	1.076	0.281
Pitch F0 (Hz)	235.79	5.45	172.15	3.21	5.256	<0.001
	**Non-vocal sound**					
RMS (dB, intensity)	65.24	0.054	65.41	0.042	2.109	0.034
Pitch F0 (Hz)	221.06	4.27	200.059	3.08	3.161	<0.001

Table S1: Statistical comparison of the mean acoustic features on the EEG time window 68-118ms where significant neurophysiological differences were found. Note that the marginally significant difference in RMS (dB, intensity) in the non-vocal sound condition is due to the small variance within the stimuli (see the near-identical values of the mean and *SEM*).
